# Intra coronary freshly isolated bone marrow cells transplantation improve cardiac function in patients with ischemic heart disease

**DOI:** 10.1186/1756-0500-5-195

**Published:** 2012-04-25

**Authors:** Ilkay Bozdag-Turan, R Goekmen Turan, Sophie Ludovicy, Ibrahim Akin, Stephan Kische, Henrik Schneider, Tim C Rehders, C Hakan Turan, Nicole S Arsoy, Tina Hermann, Liliya Paranskaya, Jasmin Ortak, Peter Kohlschein, Manuela Bastian, Kurtulus Sahin, Christoph A Nienaber, Hueseyin Ince

**Affiliations:** 1 Department of Internal Medicine, Division of Cardiology, Rostock-University, Ernst Hydemann Str 6, Rostock 18055, Germany; 2 Institute of Clinical Chemistry & Laboratory Medicine, University of Rostock, Rostock, Germany; 3 Institute for Clinical Research and Statistics, Cologne, Germany

**Keywords:** Ischemic heart disease, Freshly isolated bone marrow cell transplantation, Global EF, Infarct size

## Abstract

**Background:**

Autologous bone marrow cell transplantation (BMCs-Tx) is a promising novel option for treatment of cardiovascular disease. In this study we analyzed whether intracoronary autologous freshly isolated BMCs-Tx have beneficial effects on cardiac function in patients with ischemic heart disease (IHD).

**Results:**

In this prospective nonrandomized study we treated 12 patients with IHD by freshly isolated BMCs-Tx by use of point of care system and compared them with a representative 12 control group without cell therapy. Global ejection fraction (EF) and infarct size area were determined by left ventriculography.

Intracoronary transplantation of autologous freshly isolated BMCs led to a significant reduction of infarct size (p < 0.001) and an increase of global EF (p = 0.003) as well as infarct wall movement velocity (p < 0.001) after 6 months follow-up compared to control group. In control group there were no significant differences of global EF, infarct size and infarct wall movement velocity between baseline and 6 months after coronary angiography. Furthermore, we found significant decrease in New York Heart Association (NYHA) as well as significant decrease of B-type natriuretic peptide (BNP) level 6 months after intracoronary cell therapy (p < 0.001), whereas there were no significant differences in control group 6 months after coronary angiography.

**Conclusions:**

These results demonstrate that intracoronary transplantation of autologous freshly isolated BMCs by use of point of care system is safe and may lead to improvement of cardiac function in patients with IHD.

**Trial registration:**

Registration number: ISRCTN54510226

## Background

Cardiac performance after myocardial infarction is compromised by ventricular remodelling, which represents a major cause of late infarct-related chronic heart failure and death [1,2]. Although conventional drug therapy may delay remodelling, there is no basic therapeutic regimen available for preventing or even reversing this process. By the use of interventional therapeutics, recanalization of the occluded infarct-related artery is possible, thereby improving or normalizing coronary blood flow. However, despite sufficient reperfusion of infracted tissue, the viability of the infracted myocardium cannot, or can only insufficiently, be improved in most of these patients [3]. Cell therapy is a promising novel option to improve vascularization or cardiac regeneration [4]. In animal models, bone marrow-derived stem/progenitor cell infusion improves cardiac function and neovascularization after myocardial infarction [5-9]. Additionally, clinical trials indicate a benefical effect of intra coronary infusion of BMCs or circulating progenitor cells (CPCs) on myocardial function in patients with acute myocardial infarction (AMI) [10-14]. It is unknown whether freshly isolated BMCs transplantation have beneficially affects postinfarction remodelling. In this prospective nonrandomized control trial, we analyzed the influence of intracoronary freshly isolated cell therapy by use of point of care system on cardiac function in patients with ischemic heart disease (IHD).

## Methods

### Patients

In a prospective nonrandomized controlled trial, patients between 18–80 years of age were eligible for inclusion in this study if they had had a documented STEMI (ST-Elevation myocardial infarction) on ECG at least 3 months and had a clear-cut demarcated region of left ventricular dysfunction with an open infarct-related coronary artery at the time of stem cell therapy. Exclusion criteria were the presence of acutely decompensated heart failure with a New York Heart Association (NYHA) class of IV, infectious or inflammatory disease, active bleeding, surgery or trauma within 2 months, renal or liver dysfunction, thrombocytopenia, or anemia, alcohol or drug dependency, a history of other severe chronic diseases or cancer, or unwillingness to participate. The local ethics committee of University Rostock approved the study protocol. All the participants have given their written informed consent. All IHD patients were discharged with standard medication consisting of acetylsalicylic acid and/or clopidogrel, an ACE inhibitor, a ß-blocker and a statin.

### Study protocol

24 IHD patients were enrolled in this prospective nonrandomized controlled study. 12 of them underwent intracoronary transplantation of autologous freshly isolated BMCs by use of point of care system whereas 12 patients served as a control group who received only coronary angiography and left ventriculography without any cell based therapy. We performed in all patients of both groups before cell transplantation a coronary angiography as well as a left ventriculography and presented with open infarct-related coronary arteries. Patients included in the cell therapy group underwent a bone marrow puncture and BM aspiration on day 1 after admission. BMCs were separated. Subsequently, after coronary angiography and left ventriculography, the BMCs were freshly transplanted intracoronary. The primary end point of the study was the change in global EF as well as the size of infarcted area measured by left ventriculography after 6 months. Secondary end point was the functional status by NYHA classification and B-type natriuretic peptide (BNP) level in peripheral blood (PB) in both groups. All data were obtained by blinded expert readers unaware of patient group assignment.

### Preparation and administrations of BMC

A total of approximately 120 ml of bone marrow was aspirated from the iliac crest after local anaesthesia and mononuclear cells were isolated and identified including CD34^+^ and CD133^+^. The bone marrow cell concentrate suspension was isolated using the Harvest BMAC System (Harvest Technologies GmbH, Munich, Germany) according to the manufactures instructions for use to produce 20mls of concentrated cells. The concentrate consisted of a heterogeneous cell population including hematopoietic, mesenchymal, other progenitor cells as well as granulocytes and platelets.

After undergoing arterial puncture, all patients received 7500 to 10000 Units of heparin. Cell transplantation was performed via the intracoronary administration route [15] using four to six fractional infusions parallel to balloon inflation over 2 to 4 min of 3 to 5 ml of cell suspension. All cells were infused directly into the infarcted zone through the infarct related artery via an angioplasty balloon catheter, which was inflated at a low pressure (4 to 8 atm) and was located within the previously stented coronary segments. This prevented back flow of cells and produced stop flow beyond the site of balloon inflation to facilitate high-pressure infiltration of cells into the infarcted zone with prolonged contact time for cellular migration. 6 months after catheter-guided cell transplantation, all functional tests were repeated, including coronary angiography and left ventriculography. There were no procedural or cell-induced complications and there were no side effects in any patients.

### Coronary angiography and left ventriculography

Patients in both groups underwent left heart catheterization, left ventriculography and coronary angiography. Cardiac function, End-diastolic volume (LVEDV), End-systolic volume (LVESV), Stroke volume index (SVI) and infarct size were determined by left ventriculography. Left ventriculography was performed for each patients in 2 projections LAO 30/0° and RAO 50/0°. Cardiac function was evaluated by global EF and by auxotonic myocardial contractility index, evaluated by the wall movement velocity of the infarcted area. Global EF was measured with Quantcor software (Siemens, Erlangen/Germany). To quantify the size of infarct area we used the centreline method according to Sheehan [16] by plotting five axes perpendicular to the long axis of the heart in the main akinetic or dyskinetic segment of ventricular wall. Systolic and diastolic lengths were then measured by two blinded independent observers, and the mean difference was divided by systolic duration in seconds. The follow-up was 6 months after the treatment. All hemodynamic baseline and follow-up investigations and between the two groups were obtained in a blinded nonrandomized order by two independent observers/cardiologist.

### Safety parameters

To assess any inflammatory response and myocardial reaction after cell therapy, white blood cell count, the serum levels of C-reactive protein (CRP) and of creatine kinase (CK) were determined immediately before and after treatment. Additional analysis was done directly after transplantation and three months later: BNP level in PB, ECG at rest, 24-h Holter ECG and echocardiography. Procedural complications were defined as any ventricular arrythmia, visible thrombus formation, distal embolization, or injury of the coronary artery associated with the cell infusion catheterization procedure.

### Statistical analysis

Quantitative data are presented with mean ± SD and qualitative data are tabulated using absolute frequencies and/or percentages. Differences between therapy groups for qualitative variables were tested using Fisher's-Exact-Test due to small number of patients in therapy groups. Within differences of quantitative variables in each therapy group are compared using the Wilcoxon-Test for depending samples, and differences between therapy groups of quantitative variables are compared with the Wilcoxon-Test (Mann–Whitney-Test) for independent samples. Both of those nonparametric Wilcoxon-Tests are prefered due to the more likely expected non-normal distribution of the data. For all statistical tests, a result will be seen as statistically significant, if the corresponding two-sided p-value is smaller or equal to 0.05. If the mean and the median did not differ markedly for a variable, the graphical presentation of the data was be done using the mean and SD of this variable. Statistical analysis was performed with SPSS for Windows (Version 15.0)

## Results

### Baseline characteristics of the patients

12 patients in the intervention group received BMCs-Tx, whereas 12 patients in second group received no intra coronary BMCs-Tx and served as a control. There were no significant differences between the baseline characteristics and demographics of patients between both groups. Moreover there were also no significant differences in medical therapy at 3 and 6 months after procedure between both groups (Table 1)

**Table 1:** Baseline clinical characteristics of patients with ischemic heart disease with bone marrow cells transplantation and control group without transplantation

	**IHD with BMCs-Tx(n=12)**	**IHD without BMCs-Tx(n=12)**	**P**
Age	62 ± 6	60 ± 5	NS
m/f	8/4	9/3	NS
Cardiovascular Risk Factors (%)			
Hypertension	70	65	NS
Hyperlipidemia	60	60	NS
Smoking	80	75	NS
Diabetes	30	30	NS
Positive family history of CAD	20	15	NS
Transmural myocardial infarction, months before Tx	20 ± 8	24 ± 8	NS
No. of diseased vessels	2 ± 0.8	2 ± 0.8	NS
Infarct-related vessel (LAD/LCX/RCA)	10/0/2	9/0/3	NS
Time from symptom onset to first reperfusion therapy (hr)	6 ± 5	6 ± 4	NS
Primary reperfusion therapy (%)	100	100	NS
PTCA/Stent at the time of AMI	12/12	12/12	NS
Medication (%)			
Aspirin	100	100	NS
Clopidogrel	100	100	NS
ACE inhibitor or AT II blocker	100	100	NS
Beta-blocker	100	100	NS
Aldosterone Antagonist	25	25	NS
Statin	100	100	NS
Laboratory parameters			
CPK U/L	2010 ± 540	2145 ± 670	NS

Ischemic heart disease (IHD), Bone marrow cells transplantation (BMCs-Tx), Coronary artery disease (CAD), Percutaneous transluminal coronary angioplasty (PTCA), Creatine phosphokinase (CPK), Left anterior descending coronary artery (LAD), Left circumflex artery (LCX), Right coronary artery (RCA), None significant (NS).

### Cellular composition of point of care system from bone marrow cells

Table 2 showed the cellular composition of bone marrow aspirate (120 ml) and bone marrow concentrate (20 ml) as well as viability of cells by use of point of care system. The number of cells of total nucleated cells, CD34^+^, CD133^+^ and platelets count increased significantly post seperation in bone marrow concentrate compared to pre seperation in bone marrow aspirate (p < 0.001).

**Table 2:** The Cellular Composition of Bone Marrow Aspirate and Bone Marrow Concentrate by use of point of care system in the group with BMCs-Tx

	**Bone Marrow Aspirate(Pre Separation, 120 cc)**	**Bone Marrow Concentrate(Post Separation, 20 cc)**
Total nucleated cells (x10^6^ ml)	28 ± 8	100 ± 24
CD34^+^ cells (x10^6^ ml)	0.25 ± 0.08	0.93 ± 0.2
CD133^+^ cells (x10^6^ ml)	0.07 ± 0.006	0.37 ± 0.04
Platelet count (x10^3^/μl)	162 ± 21	697 ± 159
Viability of cells (%)	98 ± 1.5	

### Effect of freshly isolated BMCs transplantation

#### Left ventricular function, infarct size and infarct wall movement velocity

**Figure 1:**
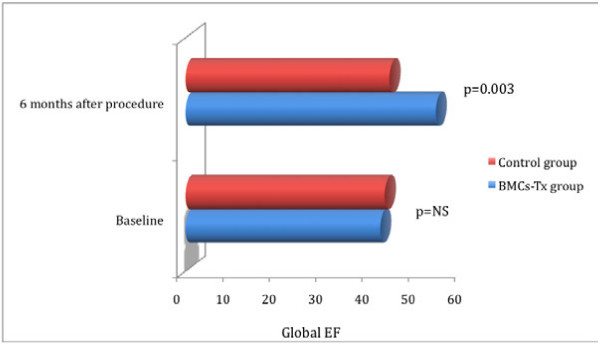
**Global EF were measured by left ventriculography immediately pre and 6 months after procedure in both groups**. There were no significant baseline differences in global EF between the two groups. Global EF significantly increased 6 months after cell therapy as compared to control group. Furthermore, no significant changes were observed in the control group at follow-up.

**Figure 2:**
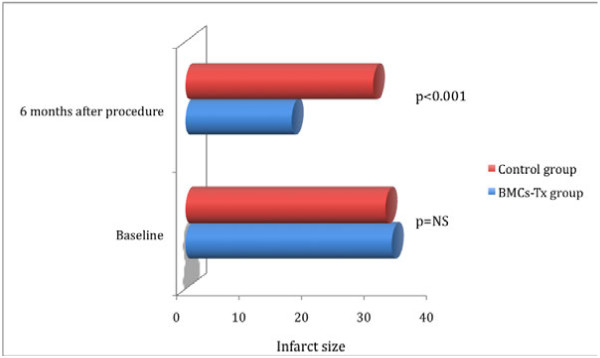
**Infarct wall movement velocity were measured by left ventriculography immediately pre and 6 months after procedure in both groups**. There were no significant baseline differences in infarct wall movement velocity between the two groups. infarct wall movement velocity significantly increased 6 months after cell therapy as compared to control group. Furthermore, no significant changes were observed in the control group at follow-up.

**Figure 3:**
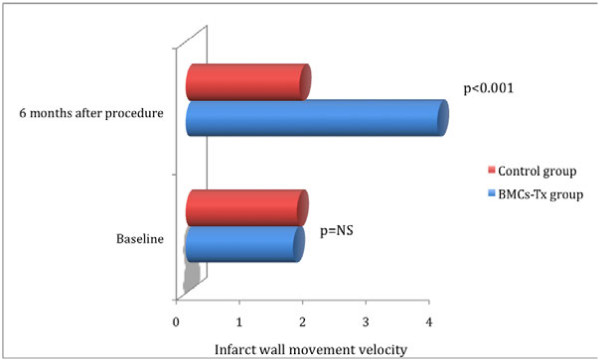
**Infarct size were measured by left ventriculography immediately pre and 6 months after procedure in both groups**. There were no significant baseline differences in infarct size between the two groups. There was a significant decrease of infarct size 6 months after cell transplantation compared to control group without cell therapy. Moreover, no significant changes were observed in the control group at follow-up.

Global EF, LVEDV, LVESV, SVI, infarct size and the wall movement velocity of the infarcted area were measured by left ventriculography in the first group immediately before and 6 months after BMCs-Tx as well as in the second group without BMCs-Tx pre- and 6 months after cardiac catheterization. There were no significant baseline differences in global EF, LVEDV, LVESV, SVI, infarct size and infarct wall movement velocity between the two groups (Tables 3 and 4). 6 months after cell therapy, we observed a significant increase of global EF and infarct wall movement velocity compared to baseline. Furthermore, we found significant decrease of infarct size after 6 months compared to baseline (Table 3). Moreover, global EF and wall movement velocity of the infracted area significantly increased 6 months after cell therapy compared to control group (Figures 1 and 2). Infarct size significantly decreased 6 months after BMCs-Tx as compared to control group without cell therapy (Figure 3). Additionally we found a significant increase of SVI and decrease of LVESV whereas no significant change was observed in LVEDV 6 months after cell therapy (Table 3). In the control group there were no significant changes in global EF, LVEDV, LVESV, SVI, infarct size and the wall movement velocity of the infarcted area 6 months after coronary angiography (Table 4).

**Table 3:** Cardiac function, clinical parameter immediately pre- and 6 months after bone marrrow cells transplantation in the bone marrow cell transplantation group

	**Immediately pre BMCs-Tx**	**6 months after BMCs-Tx**	**P**
Global EF (%)	42 ± 8	54 ± 10	p = 0.001
The size of infarct area (%)	33 ± 10	17 ± 10	p < 0.001
Infarct wall movement velocity (cm/s)	1.70 ± 0.90	3.98 ± 0.86	p < 0.001
End-diastolic volume (LVEDV) (ml)	128 ± 45	136 ± 50	p = NS
End-systolic volume (LVESV) (ml)	74 ± 25	63 ± 25	p < 0.01
Stroke volume index (SVI) (ml/m^2^)	30 ± 10	40 ± 8	p < 0.01
BNP (pg/ml)	169 ± 85	72 ± 21	p < 0.001
NYHA classification	II-III	I-II	p < 0.001

**Table 4:** Cardiac function, clinical parameter pre- and 6 months after coronary angiography in control group without bone marrrow cells transplantation

	**Immediately pre coronary angiography**	**6 months after coronary angiogrpahy**	**P**
Global EF (%)	43 ± 10	44 ± 8	p = NS
The size of infarct area (%)	32 ± 10	30 ± 10	p = NS
Infarct wall movement velocity (cm/s)	1.76 ± 0.76	1.80 ± 0.76	p = NS
End-diastolic volume (LVEDV) (ml)	132 ± 35	136 ± 48	p = NS
End-systolic volume (LVESV) (ml)	75 ± 29	76 ± 29	p = NS
Stroke volume index (SVI) (ml/m^2^)	29 ± 11	33 ± 15	p = NS
BNP (pg/ml)	155 ± 71	141 ± 69	p = NS
NYHA classification	II-III	II-III	p = NS

Values are mean ± SD. New York Heart Association (NYHA), Global ejection fraction (Global EF), B-type natriuretic peptide (BNP), Non significant (NS).

#### Functional status and clinical safety parameters

**Figure 4:**
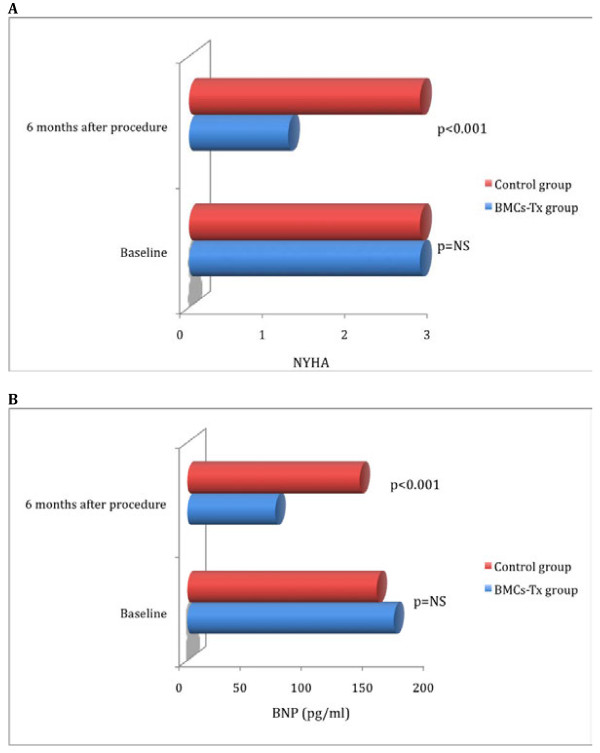
**NYHA classification and BNP levels in both groups.** There were no significant differences of baseline NYHA classification and BNP levels between two groups. 6 months after cell therapy there were a significant decrease of NYHA classification and BNP levels compared to control group without cell therapy. Moreover, no significant changes were observed in the control group at follow up.

To determine the functional status we assessed NYHA classification and BNP levels in both groups by two independent and blinded physicians. There were no significant differences of at baseline NYHA classification and BNP levels between both groups. We observed significant decrease in NYHA classification and BNP levels 6 months after intracoronary cell therapy, whereas there were no significant differences in control group 6 months after coronary angiography (Table 3 and 4). NYHA classification and BNP levels significantly decreased 6 months after cell therapy compared to control group (Figure 4).

ECG at rest, on exercise and 24-h Holter ECG revealed no rhythm disturbances. There was no inflammatory response or myocardial infarction (white blood cell count, CRP, CK) after cell therapy. No immediate periprocedure as well as postprocedure adverese complications and no new electrocardiographic changes or significant elevations in CK or troponin as well as no inflammatory response were observed in patients with BMC-Tx

## Discussion

In this prospective nonrandomized controlled study we could demonstrate improved global EF, infarct size as well as functional status after intra coronary autologous freshly isolated bone marrow cells transplantation in patients with IHD.

Despite improved pharmacological therapy, congestive heart failure remains the leading cause of cardiovascular mortality in industrialized world [17]. The use of stem cell-based therapy is becoming increasingly recognized as having the potential to salvage damaged myocardium and to promote endogenous repair of cardiac tissue, thus having the potential for the treatment of heart failure. Experimental studies demonstrated that intravascular or intramyocardial administration of BMCs or CPCs may contribute to functional regeneration of infarcted myocardium and enhance neovascularization of ischaemic myocardium [5-9]. Clinical pilot and randomized trials suggested, that the intracoronary infusion of autologous BMCs is safe and feasible as well as beneficially affects left ventricular contractile recovery and infarct size in patients with AMI [10-14]. The beneficial effects observed in most phase I/II studies were confirmed in the so far largest douple-blind, randomized multicenter REPAIR-AMI trial [14]. Only one larger study, the ASTAMI trial [18] did not show any benefit on leftventricular functional parameters. The reason for the failure of the ASTAMI trial to show a benefit of cell therapy may have been the different cell isolation and storage protocol, which significantly affected the functional capacity of the cells [19]. Likewise, it was reported, that intracoronary infusion of BMCs in patients with ischemic heart disease improve moderate left ventricular function [20,21]. In our study, we demonstrated that the infarct size reduced, whereas the global EF and regional infarct wall movement velocity increased 6 months after intracoronary cell therapy in patients with IHD. This observation is in line with the data of Strauer et al [20]. and Assmus et al [21]. In addition, we observed decrease in NYHA classification and BNP levels 6 months after cell therapy. Because most of the previous clinical trials involved BMCs isolated by Ficoll [10-14], this thecnique currently viewed as the gold standard. Cell isolation procedures are crucial for the functional activity of the administered cellular product. In our trial we chose to use a point of care system for the preparation of the treating cell composition. We showed in pilot study that freshly isolated BMCs-Tx by use a point of care system is safe and feasible as well as may improve the cardiac function also in patients after AMI [22]. Previous studies have demonstrated, that the mobilization and functional activity of CD34/45^+^ and CD133/45^+^ BM-CPCs significantly increased after intracoronary infusion of BMCs in patients with ischemic heart disease [23,24]. We demonstrated the same results for the first time with intracoronary freshly isolated BMCs-Tx by use a point of care system in patients with IHD, not Ficoll gradient separation as in other studies. Unlike many previously conducted trials that employed Ficoll gradient separation as the method of cell collection, which produces a very limited cell linage spectrum. The cellular composition of the concentrate, which was prepared by use a point of care system, differs from that prepared using the Ficoll method. The Ficoll composition contains predominantly mononuclear cells (lymphocytes, erythroblasts and monocytes) and very few granulocytes. The point of care system concentrates entire nucleated cell population with mononuclear cells and specific stem cell population (CD34+ and CD133+) as well as the platelets from the marrow aspirate (Table 2). Importantly, however, the point of care device provided advantage of significantly higher yield of isolated bone marrow cells compared to the Ficoll protocol. Thus, if the number of infused cells in invivo neovascularization model was adjusted for this higher yield of bone marrow cells, the treatment effect was significantly greater compared to Ficoll BMCs, as assessed by limb perfusion measurement [25]. One obvious difference in the two compositions is the presence of significant numbers of granulocytes and platelets in the point of care system composition. Platelets and granulocytes have been shown to have a positive effect on the neovascular potential of the resulting concentrate. The presence of platelets within composition could be important because it has been shown that these platelet-derived mediators also potently enhance postnatal angiogenesis. Iba and colleagues demonstrated that implantation of mononuclear cells together with platelets into ischemic limbs more effectively augments collateral vessel formation by supplying various angiogenic factors, in which VEGF played a key role [26]. Indeed, Massberg and colleagues provided compel ling evidence that platelets generate the critical signal that recruits CD34+ bone marrow cells and c-Kit + Sca-1+ Lin- bone marrow-derived progenitor cells to sites of injury [27]. Therefore, these findings strongly support the notion that implanted platelets play a pivotal role in stem and progenitor recruitment and provide a rationale for the fact that point of care system produced functional in vivo results similar to or better than Ficoll. In our study despite higher number of platelets we observed no immediate periprocedure as well as postprocedure adverese complications. In addition, unlike Ficoll isolation where cells are resuspended in a serum free medium, point of care system is always resuspended in the patient’s own plasma. Thus, the isolated cells are not removed from their natural plasma microenvironment, which may be help to sustain the functionality of the cells. This has been further supported by experimental study of Hermann et al., who showed that the point of care system composition to be significantly more bioactive than the Ficoll composition. Intriguingly, however, due to the greater yield of cells generated by use a point of care system, the cellular product isolated from a given bone marrow aspirate by use a point of care device may actually translate into even greater therapeutic effects. Additionally, practical aspects may also deserve consideration. Importantly, a major limitation of the Ficoll isolation procedure for clinical applications is that it is strongly investigator dependant, immensely time consuming and requires a good manufacturing practice (GMP) facility. In this study we were able to demonstrate that such complex methods are not necessary to achieve established results. As the concentration process by use of point of care system, everything can be accomplished in one session without adding excessive time to the overall procedure circumventing the previously mentioned disadvantages of the Ficoll isolation process. The point of care device provides a much shorter turnaround time. Therefore, this device represents a cost-effective and time-efficient stand-alone technique for the isolation of autologous bone marrow cells suitable for cell therapy regimens in the rapidly growing field of regenerative medicine.

Several hypotheses have been proposed about, how intracoronary cell therapy improves myocardial function. I) Experimental studies addressing the capacity of transplanted bone marrow-derived stem cells to differentiate into the cardiomyogenic lineage yielded conflicting results. Recent well-conducted studies suggest that the BMCs do not transdifferentiate into cardiomyocytes but adopt mature hematopoeitic characteristics. In contrast to embryonic stem cells, most adult stem or progenitor cells do not spontaneously differentiate into cardiomyocytes but rather require an adequate stimulus to do so. II) Another proposed mechanism is that cell therapy may increase angiogenesis and improve blood supply to ischemic regions, potentially aiding in the revascularization of hibernating myocardium and inhibiting cardiomyocyte apoptosis. Additionally or alternatively, the local microenvironment plays an important role to induce cell fate changes by physical cell-to-cell interaction or by providing paracrine factors promoting tissue repair [28-30].

Cell-based therapy is a promising option for treatment of ischemic disease. However, cell therapy is in its early stages, and various questions remain. BMCs are best characterized and have been used in the majority of clinical trials performed to date. BMCs contains a complex assortment of progenitor cells, including hematopoietic stem cells (HSCs), mesenchymal stem cells (MSCs) or stromal cells and multipotential adult progenitor cells (MAPCs) [31]. Additionally, The presence of immature circulating cells in the peripheral blood has been advocated as a marker of an organism`s regenerative capacity [32].

The primary limitation of this study is the only use of left ventriculography for cardiac function without the additionally measurement of cardiac imaging. However the same assessments was used in several studies [10,14,15,20,21]. Moreover, for better understanding we added left ventricular volume data in our study. Secondly the placebo effect on improvement of NYHA classification in cell therapy group can not be excluded due to the obvious. On the other hand this result was confirmed indirectly by decrease of BNP values in PB and improvement of global EF. Therefore, a randomized placebo-controlled study will be needed to validate the hypothesis.

## Conclusions

In the present study we could demonstrate that intracoronary transplantation of autologous freshly isolated BMCs by point of care system improved global EF and reduced infarct size significantly in patients with IHD after 6 months. Moreover, we observed a significant decrease of NYHA-classification and BNP levels even 6 months after cell transplantation. This interesting observation could be implemented in future large-scale randomized studies.

## Abbreviations

BMCs-Tx, Autologous freshly isolated bone marrow cells transplantation; IHD, Ischemic heart disease; EF, Ejection fraction; CPCs, Circulating progenitor cells; AMI, Acute myocardial infarction; NYHA, New York Heart Association; PB, Peripheral blood; BNP, B-type natriuretic peptide; LVEDV, End-diastolic volume; LVESV, End-systolic volume; SVI, Stroke volume index; CRP, C-reactive protein; CK, Creatine kinase.

## Competing Interest

The authors declare that they have no competing interests.

## Authors’ Contributions

All authors listed have contributed sufficiently to the project to be included as authors. All authors contributed to the acquisition and analysis/interpretation of data, as well as the conception and design.
